# Strategy for Development of Site-Specific Ubiquitin Antibodies

**DOI:** 10.3389/fchem.2020.00111

**Published:** 2020-02-21

**Authors:** Ila van Kruijsbergen, Monique P. C. Mulder, Michael Uckelmann, Tibor van Welsem, John de Widt, Aldo Spanjaard, Heinz Jacobs, Farid El Oualid, Huib Ovaa, Fred van Leeuwen

**Affiliations:** ^1^Division of Gene Regulation, Netherlands Cancer Institute, Amsterdam, Netherlands; ^2^Leiden Institute for Chemical Immunology, Oncode Institute, Leiden University Medical Center, Leiden, Netherlands; ^3^Division of Biochemistry, Netherlands Cancer Institute, Amsterdam, Netherlands; ^4^Division of Tumor Biology & Immunology, Netherlands Cancer Institute, Amsterdam, Netherlands; ^5^UbiQ Bio BV, Amsterdam, Netherlands; ^6^Department of Medical Biology, Amsterdam UMC, University of Amsterdam, Amsterdam, Netherlands

**Keywords:** ubiquitin, histone H2B, H2B-K123ub, PCNA, monoclonal antibody

## Abstract

Protein ubiquitination is a key post-translational modification regulating a wide range of biological processes. Ubiquitination involves the covalent attachment of the small protein ubiquitin to a lysine of a protein substrate. In addition to its well-established role in protein degradation, protein ubiquitination plays a role in protein-protein interactions, DNA repair, transcriptional regulation, and other cellular functions. Understanding the mechanisms and functional relevance of ubiquitin as a signaling system requires the generation of antibodies or alternative reagents that specifically detect ubiquitin in a site-specific manner. However, in contrast to other post-translational modifications such as acetylation, phosphorylation, and methylation, the instability and size of ubiquitin−76 amino acids–complicate the preparation of suitable antigens and the generation antibodies detecting such site-specific modifications. As a result, the field of ubiquitin research has limited access to specific antibodies. This severely hampers progress in understanding the regulation and function of site-specific ubiquitination in many areas of biology, specifically in epigenetics and cancer. Therefore, there is a high demand for antibodies recognizing site-specific ubiquitin modifications. Here we describe a strategy for the development of site-specific ubiquitin antibodies. Based on a recently developed antibody against site-specific ubiquitination of histone H2B, we provide detailed protocols for chemical synthesis methods for antigen preparation and discuss considerations for screening and quality control experiments.

## Introduction

The covalent attachment of ubiquitin (Ub) to proteins constitutes a key post-translational modification mechanism. Ubiquitin, a polypeptide of 76 amino acids, is perhaps best known for its role in protein degradation (Rosenbaum et al., 2011; Claessen et al., 2012; Geng et al., 2012; Komander and Rape, 2012; Varshavsky, 2012). However, more recently ubiquitin has also emerged as a powerful and versatile signaling system that regulates many different biological pathways (Chen and Sun, 2009; Husnjak and Dikic, 2012; Ramanathan and Ye, 2012; Oh et al., 2018; Rape, 2018; Clague et al., 2019; Mattern et al., 2019; Spit et al., 2019). Ubiquitin can influence protein-protein interactions, protein targeting and sorting, and regulates processes such as gene expression, and DNA repair (Ranjitkar et al., 2010; Piro et al., 2012; Marteijn et al., 2014; van Cuijk et al., 2014; Zhao et al., 2014; Venkatesh and Workman, 2015). Ubiquitin impinges on many critical processes in the cell and thereby plays major roles in normal development and human aging and disease. The molecular mechanisms involved are topics of intensive research, both in basic science as well as in the clinic (Chen and Sun, 2009; Geng et al., 2012; Mattiroli and Sixma, 2014).

Ubiquitin-mediated protein degradation typically involves the attachment of poly-ubiquitin chains (poly-Ub) to target proteins (Varshavsky, 2012). Here, ubiquitin moieties are attached to each other via a lysine 48 (K48) residue of another ubiquitin molecule. This process has been studied extensively and is relatively well-understood because monitoring poly-ubiquitination requires simple non-specific detection methods and can be easily modulated by inhibition of the proteasome, the molecular machine that degrades K48-poly-ubiquitylated proteins. In contrast, the understanding of other signaling- and degradation-functions of ubiquitin is much less developed because of the lack of specific reagents required to study them. Different ubiquitination patterns involve the attachment of one ubiquitin molecule (Ub1) to a specific lysine residue of a target protein or the attachment of a poly-ubiquitin chain involving homogeneous or mixed chains by forming isopeptide bonds between the N-terminal methionine (Met1-linked ubiquitination) or any of the other internal lysines on ubiquitin (Lys6, Lys11, Lys27, Lys29, Lys33, Lys63) (Chen and Sun, 2009; Husnjak and Dikic, 2012; Ramanathan and Ye, 2012; Oh et al., 2018; Rape, 2018; Clague et al., 2019; Mattern et al., 2019; Spit et al., 2019). Efficient monitoring of these types of events requires reagents that specifically recognize the attachment of ubiquitin to one particular lysine residue of a protein or of ubiquitin itself (poly-Ub) (Fujimuro et al., 1994; Fujimuro and Yokosawa, 2005; Newton et al., 2008, 2012; Matsumoto et al., 2010, 2012; Fulzele and Bennett, 2018; Mattern et al., 2019; van Wijk et al., 2019). To date, very few reagents exist that fulfill these criteria. Whereas many antibodies have been developed to detect a range of known protein modifications (e.g., acetylation, methylation, phosphorylation), generation of antibodies against site-specific ubiquitination appears to be far from trivial. As a result, a limited number of suitable antibodies exists that allow for the monitoring and studying of specific ubiquitination events.

The development of site-specific ubiquitination antibodies faces several critical challenges due to the large size and reversibility of the ubiquitin modification. First, whereas a methylated lysine or phosphorylated serine can be easily incorporated into synthetic peptides by standard synthesis methods, incorporation of a ubiquitinated lysine requires advanced chemistry. As a way out, short fragments of ubiquitin have been used in immunization strategies. However, as evidenced by the small number of good antibodies this approach has not always been successful. Second, ubiquitin is covalently attached to lysine residues in proteins but can be readily removed by deubiquitinating enzymes that are also present in plasma, likely as a result of leakage from dead cells. Therefore, the native ubiquitin-lysine isopeptide linkage is likely to be cleaved upon immunization.

To solve these problems, we applied synthesis of full-length ubiquitin and derivatives thereof that can be attached to target peptides of choice and applied chemical ligation technologies that allow synthesis of well-defined Ub-modified polypeptides, either with a native isopeptide linkage using thiolysine mediated ligation or with a proteolytically stable bond using click chemistry. In the latter case the overall structure around the native Ub-Lysine environment is preserved to a maximum extent by only replacing the native isopeptide bond between Ub and the lysine residue with a proteolytically stable amide triazole isostere. This enables the use of immunization antigens that closely resemble the isopeptide linked Ub conjugate. The triazole isostere has been shown to be a good amide-bond mimic that is tolerated and has been utilized in biological settings involving triazole-based poly-Ub chains as well as in activity-based probes (Weikart et al., 2012; Dresselhaus et al., 2013; McGouran et al., 2013; Flierman et al., 2016). The introduction of the whole ubiquitin protein in a proteolytically stable form is expected to increase the chance of exposing a site-specific epitope for generating high quality antibodies. This idea is supported by the recent success of a monoclonal antibody specific for ubiquitin on lysine 123 of yeast histone H2B (yH2B-K123ub1), which was obtained using this approach (van Welsem et al., 2018; Vlaming et al., 2019). H2BK123ub in yeast or H2BK120 in metazoans is known to play key roles in regulating gene expression, chromatin dynamics, and DNA repair (Fuchs and Oren, 2014; Cole et al., 2015; Morgan and Wolberger, 2017; Marsh and Dickson, 2019; Worden and Wolberger, 2019). The antibody has successfully been used for immunoblots and chromatin immunoprecipitation assays (Vlaming et al., 2016, 2019; van Welsem et al., 2018) and thereby enabled the deconstruction of a bidirectional regulatory mechanism between histone methylation and histone ubiquitination (Vlaming et al., 2016, 2019; van Welsem et al., 2018).

**Figure 1:**
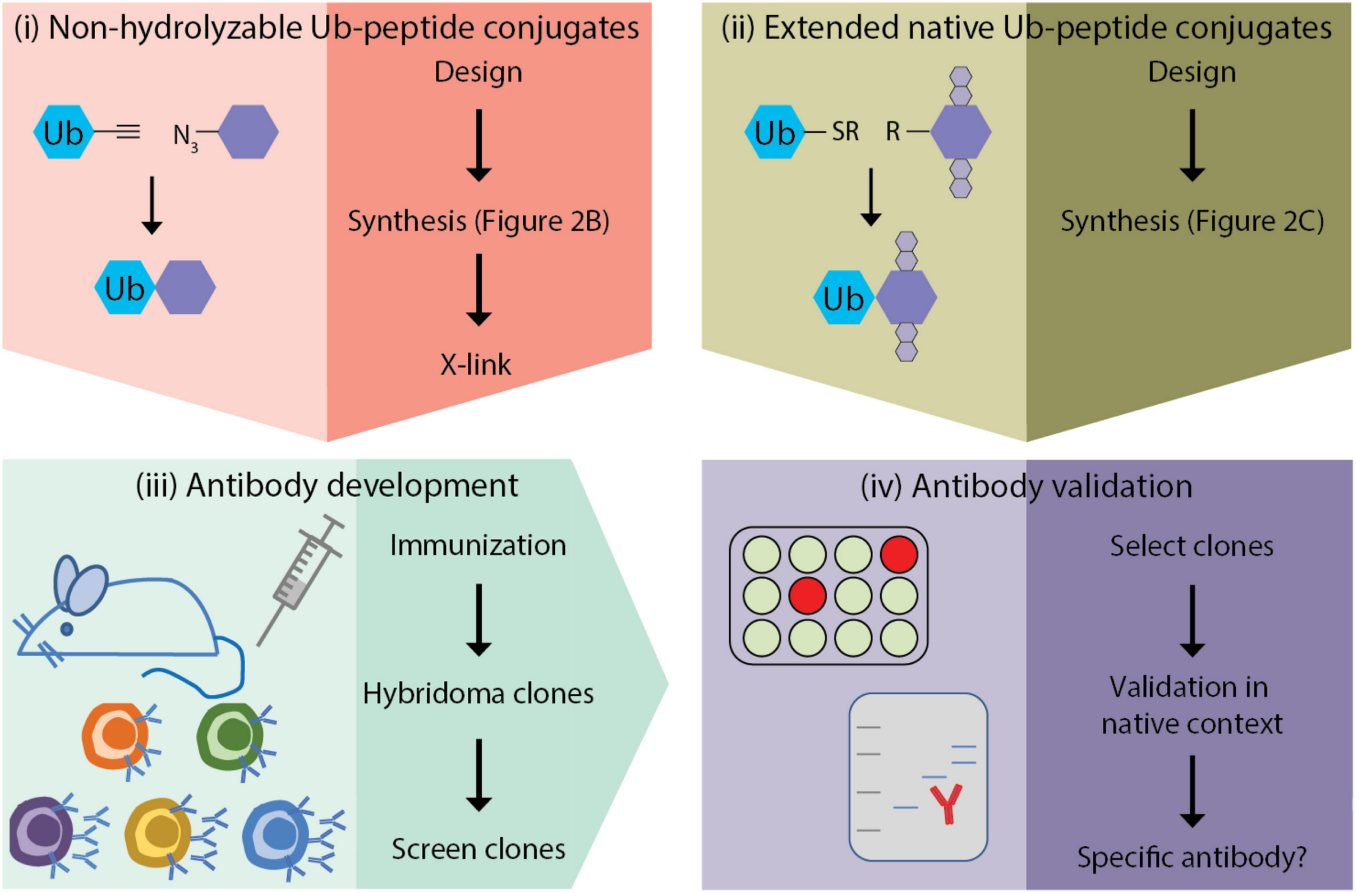
Outline of site-specific Ub antibody development. **(i)** design and synthesis of non-hydrolyzable Ub-peptide conjugates for immunization; **(ii)** design and synthesis of extended native iso-peptide linked Ub-peptide conjugates for screening; **(iii)** immunization, and generation and selection of hybridomas; **(iv)** selection of clones and antibody validation.

The generation of monoclonal antibodies in mice involves multiple steps: (i) design and synthesis of non-hydrolyzable Ub-peptide conjugates for immunization; (ii) design and synthesis of extended native iso-peptide linked Ub-peptide conjugates for screening; (iii) immunization, and generation and screening of hybridomas; (iv) clone selection and antibody validation in native context (Figure 1). In this manuscript we focus on the steps that are specific for synthesis of ubiquitin-peptide conjugates for the generation of site-specific ubiquitin antibodies (steps i and ii), as detailed excellent general protocols for antibody development (step iii) and validation (step iv) have been described elsewhere (Egelhofer et al., 2010; Yokoyama et al., 2013; Greenfield, 2014; Ossipow and Fischer, 2014; Kungulovski et al., 2015; Marcon et al., 2015; Rothbart et al., 2015; Uhlen et al., 2016; Guillemette et al., 2017; Holzlöhner and Hanack, 2017; Edfors et al., 2018; Venkataraman et al., 2018; Weller, 2018; Marx, 2019). In addition, we discuss the rationale for the design of antigens used for immunization and screening. Finally, we provide examples of Ub-specific screening and validation assays and discuss possible pitfalls based on the immunization scheme to obtain site-specific antibodies against yeast H2B-K123ub (van Welsem et al., 2018; Vlaming et al., 2019) and attempts to develop antibodies specific for lysine 164 of human proliferating cell nuclear antigen (huPCNA-K164ub). Modification of PCNA by ubiquitin critically regulates the function of PCNA in DNA damage tolerance (Hoege et al., 2002; Mailand et al., 2013; Kanao and Masutani, 2017; Uckelmann and Sixma, 2017).

Given the urgent need for site-specific ubiquitin antibodies in the field, our protocol and design strategies will be of great help to other researchers in the field of small-protein modifications and could facilitate the production of these reagents, thereby enabling insights into the dynamics of ubiquitination.

## Materials and Equipment

In this section we describe the reagents and equipment required for the synthesis of Ub-conjugates. In section Methods we describe the synthesis methods and the rationale of the conjugate design. In section Anticipated Results we show how the conjugates are used for screening and clone selection in steps and we provide examples of validation experiments for step.

### Reagents and Equipment Used for Ub-Conjugate Synthesis and Analysis

#### Reagents for Conjugate Synthesis

Fmoc-amino acids were purchased from Novabiochem, solvents, N,N-Diisopropylethylamine (DiPEA), and acetic anhydride from Biosolve, peptide coupling reagents Benzotriazol-1-yl-oxy-tripyrrolidinophosphonium hexafluorophosphate (PyBOP), 2-(1H-Benzotriazole-1-yl)-1,1,3,3-tetramethylaminium hexafluorophosphate (HBTU) and Hydroxybenzotriazole (HOBt) from Novabiochem (Merck).

#### Equipment and Software for Ub-Conjugate Synthesis and Analysis

Syro II MultiSyntech Automated Peptide synthesizer

Waters 2795 Separation Module (Alliance HT)

Waters 2996 Photodiode Array Detector (190–750 nm)

Phenomenex Kinetex C18 (2.1 × 100, 2.6 μm) column

LCTTM Orthogonal Acceleration Time of Flight Mass Spectrometer

Waters Atlantis T3 C18 (30 × 250 5 μm)

Waters Mass Lynx Mass Spectrometry Software 4.1.

### Reagents Used for Ub-Conjugate Crosslinking

#### General Reagents

- KLH (Pierce 77600 Imject mcKLH)
- BCP (Pierce 77130 Imject Blue Carrier Protein)
- Glutaraldehyde solution 25%, EM grade (Sigma G5882).

#### Antibodies Used in This Study

- yH2BK123ub1 (mouse monoclonal #152107, Ximbio) RRID: AB_2737407
- yH2BK123ub1 (non-specific rabbit polyclonal serum; #NKI-162611, this study)
- yH2B (rabbit polyclonal; #39238, Active Motif) RRID: AB_2631110
- Ubiquitin (mouse monoclonal; #NKI-28B8A12/D7, this study)
- PCNA-K164ub (non-specific mouse monoclonal; #NKI-1G5B7/F10, this study)
- PCNA (mouse monoclonal; #PC10/Sc-56, lot I0710, Santa Cruz)
- PCNA-K164ub [rabbit monoclonal; #D5C7P, lot 11/2014, Cell Signaling Technology (CST)]
- H2A (rabbit polyclonal serum; #39235, Active Motif) RRID: AB_2687477
- H4 (rabbit monoclonal; #05-858, clone 62-141-13, lot: 2459608, Millipore).

## Methods

### Rationale Antigen Design

**Figure 2:**
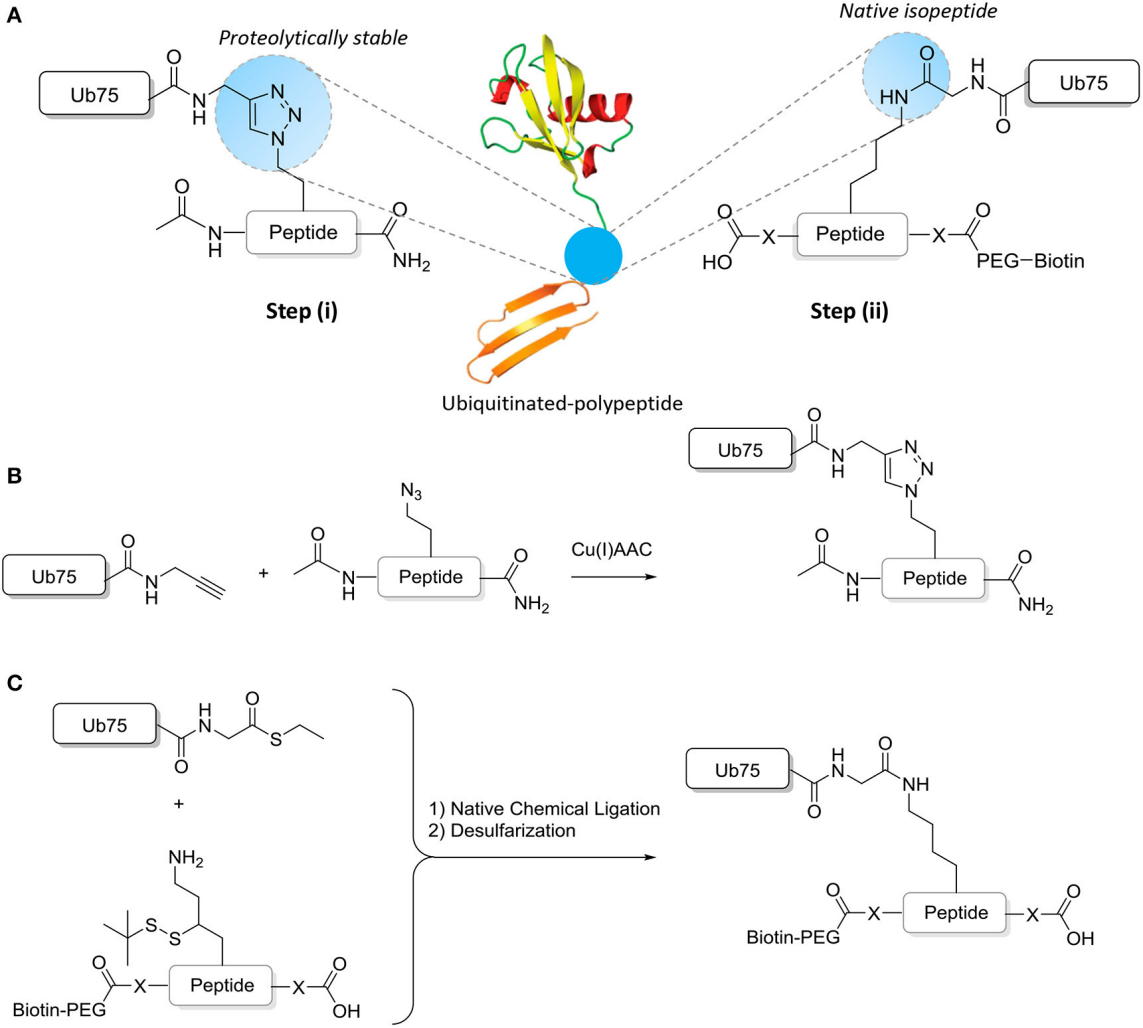
Synthesis of ubiquitinated polypeptides. **(A)** Design of the proteolytically-stable and native isopeptide-linked ubiquitinated polypeptides; **(B)** Schematic representation of the key CuAAC reaction to generate non-hydrolyzable Ub-polypeptides; **(C)** Schematic representation of the key steps to generate isopeptide linked Ub-polypeptides in which the sequence used in panel B is extended (indicated by -X- and explained further in the text).

Chemical ligation methods are used to synthesize well-defined Ub-modified polypeptides of ~15–17 amino acids either with in the central residue a native isopeptide or proteolytically stable Ub bond (Figure 2). Proteolytically-stable Ub-polypeptide conjugates are used for the initial immunization. If the peptide corresponds to an internal sequence, immunization peptides are acetylated at the N-terminus to eliminate the positively charged N-terminal amino group, which is often erroneously recognized by antibodies raised against short peptides. Similarly, the C-terminus of the peptide is amidated. ELISA screens are performed with native iso-peptide linked Ub- polypeptides that are two amino acids longer at the N-terminus and/or C-terminus (depending on the position of the ubiquitination site in the protein) than the immunization antigen to more closely mimic the native protein (as discussed below and see Figure 2). When using mice, a complete 10-mouse immunization scheme, clone-selection, and screening protocol together typically requires 4 mg Ub conjugate. For the negative control ELISA screens, 4 mg non-modified peptide and 4 mg free Ub is required per protocol. Immunizations in larger animals typically require more material.

### General Methods

LC-MS measurements are performed on a system equipped with a Waters 2795 Separation Module (Alliance HT), Waters 2996 Photodiode Array Detector (190–750 nm), Phenomenex Kinetex C18 (2.1 × 100, 2.6 μm) column and LCTTM Orthogonal Acceleration Time of Flight Mass Spectrometer. Samples are run using 2 mobile phases: A (0.1% formic acid in H_2_O/acetonitrile 99:1 v/v) and B (0.1 % formic acid in H_2_O/acetonitrile 99:1 v/v) at a flow rate of 400 μL/min; gradient: 0–0.5 min, 5% B; 0.5–8 min, → 95% B; 8–10 min 95% B, 10–12 min, → 5% B. Data processing is performed using Waters Mass Lynx Mass Spectrometry Software 4.1 (deconvolution with Maxent1 function).

### General Purification Procedures

The crude product is dissolved in a minimal amount of warm DMSO and then diluted by addition to MilliQ water and purified by RP-HPLC on a Waters Atlantis T3 C18 30 × 250 5 μm. Column Mobile phases: A = 0.05% aq. trifluoroacetic acid (TFA) and B = 0.05% TFA in CH_3_CN. Ub peptides are purified with a gradient: 20 → 45% B over 25 min, while the short polypeptides (15–17 AA) are purified with a gradient: 5 → 95% B over 25 min, Flow rate = 37 mL/min. Pure fractions (>95%), as judged by LC-MS are pooled, lyophilized and used as such.

### Synthesis of Mono-Ubiquitin Precursors

#### Ub

The Ub(1–75) and Ub(1–76) peptide sequences are synthesized on resin following the procedures described before (El Oualid et al., 2010; Mulder et al., 2014). In brief, (Ub) is synthesized on a Syro II MultiSyntech Automated Peptide synthesizer using standard 9-fluorenylmethoxycarbonyl (Fmoc) based solid phase peptide chemistry on a 25-μmol scale. Starting with the pre-loaded Fmoc-Gly trityl resin (0.18 mmol/g, Rapp Polymere GmbH), each successive amino acid (Novabiochem) is double coupled in 4 molar excess using PyBOP (4 equiv) and DiPEA (8 equiv) as coupling regents. The N-terminal methionine residue in the sequence is replaced by the known isostere Norleucine. Deprotection of the Fmoc group is achieved with 20% piperidine in N-Methyl-2-pyrrolidone (NMP) (2 × 2 and 1 × 5 min). After completion of all coupling cycles, the N-terminus is protected with a Boc group by overnight treatment with Boc-anhydride in Dichloromethane (DCM). The resin is rinsed with DCM (3 × 5 mL) and treated with 5 mL of DCM/ Hexafluoroisopropanol (HFIP) (4:1 v/v) for 30 min and filtered. The resin is rinsed with DCM (3 × 5 mL) and the combined filtrates are concentrated to obtain the partially protected Ub peptide 1–75.

#### UbPA

The partially protected peptide residue Ub(1–75) (1 equiv) is redissolved in DCM and reacted with PyBOP (5 equiv), propargyl amine (10 equiv) and triethylamine (TEA) (20 equiv). The reaction mixture is stirred over night at room temperature. After removal of the solvent *in vacuo*, the residue is treated with TFA/H_2_O/iPr_3_SiH/phenol (90/5/2.5/2.5 v/v/v/v, 1 mL for 5 μmol Ub(1–75)-PA) for 3 h followed by precipitation with cold Et_2_O/pentane (3/1 v/v). Purification by HPLC gives Ub-PA as a white powder.

#### UbSEt

The partially protected peptide residue Ub(1–76) (1 equiv) is redissolved in DCM and reacted with pyBOP (5 equiv), EtSH (10 equiv) and DiPEA (10 equiv). The reaction mixture is stirred overnight at room temperature. The solvent is removed in vacuo and the residue treated for 3 h with TFA/H_2_O/iPr_3_SiH/phenol (90/5/2.5/2.5 v/v/v/v, 1 mL for 5 μmol UbSEt) followed by precipitation with cold Et_2_O/pentane (3/1 v/v). Purification by HPLC gives UbSEt as a white powder.

### Synthesis of Peptide Precursors

#### Synthesis of Azidoornithine Peptide Precursor for Synthesis Non-hydrolyzable Conjugates

The peptides are synthesized on a Syro II MultiSyntech Automated Peptide synthesizer using standard Fmoc based solid phase peptide chemistry on a 100-μmol scale. Starting with pre-loaded resin for Fmoc Peptide synthesis (Rapp Polymere GmbH), each successive amino acid (Novabiochem) is double coupled (2 × 25 min, in the event of difficult coupling extended to 2 × 70 min) in 4 molar excess using PyBOP (4 equiv) and DiPEA (8 equiv) as coupling regents. Deprotection of the Fmoc group is achieved with 20% piperidine in NMP (2 × 5 min). After completion of all coupling cycles, the resin is treated with acetic anhydride to acetylate the N-terminus or modified with a biotin-PEG moiety to facilitate Surface Plasmon Resonance (SPR) measurements. In the latter case, a PEG spacer (8-Fmoc-amino)-3,6-dioxaoctanoic acid (AK Scientific, Inc., Union city, CA, 4 equiv), is coupled to the N-terminus using PyBOP (4 equiv) and DIPEA (4 equiv) in NMP for 25 min. at ambient temperature. The Fmoc protection group is removed as described and biotin is coupled subsequently using HBTU (2-(1H-benzotriazol-1-yl)-1,1,3,3-tetramethyluronium hexafluorophosphate, 4 equiv), HOBt (1-hydroxybenzotriazole, 4 equiv), DIPEA (8 equiv), and carboxy-functionalized biotin (Sigma-Aldrich, 4 equiv) in NMP and reacted for 2.5 h. After removal of the solvent *in vacuo*, the residue is treated for 3 h with TFA/H_2_O/iPr_3_SiH/phenol (90/5/2.5/2.5 v/v/v/v, 1 mL for 5 μmol peptide) followed by precipitation with cold Et_2_O/pentane (3/1 v/v). Purification by HPLC gives the peptide as a white powder.

In the synthesis of immunization peptides, the lysine is replaced for an azidoornithine (azOrn) moiety and the choice for solid support is based upon the peptide sequence. Peptides corresponding to an internal protein sequence, as for the PCNA-K164 peptide (UniProtKB - P12004, AA 156-172: ac-DAVVISCAazOrnDGVKFSAS-CONH_2_), are synthesized on a preloaded rink-amide resin. While peptide sequences corresponding to the C-termini of the protein, as for the H2B-K123 peptide (UniProtKB - P02294, AA 115–130: ac-SEGTRAVTazOrnYSSSTQA-COOH), are synthesized on a preloaded PEG-polystyrene support resin (PEG-PS).

#### Synthesis of γ-Thiolysine Peptide Precursor for Synthesis Native Conjugates

The peptides are synthesized on a Syro II MultiSyntech Automated Peptide synthesizer using standard Fmoc based solid phase peptide chemistry on a 100-μmol scale. Starting with pre-loaded PEG-polystyrene support resin (PEG-PS) for Fmoc Peptide synthesis (Rapp Polymere GmbH), each successive amino acid (Novabiochem) is double coupled (2 × 25 min, in the event of difficult coupling extended to 2 × 70 min^*^) in 4 molar excess using PyBOP (4 equiv) and DiPEA (8 equiv) as coupling regents. Deprotection of the Fmoc group is achieved with 20% piperidine in NMP (2 × 5 min). After completion of all coupling cycles the resin is treated with acetic anhydride to acetylate the N-terminus. After removal of the solvent *in vacuo*, the residue is treated for 3 h with TFA/H_2_O/iPr_3_SiH/phenol (90/5/2.5/2.5 v/v/v/v, 1 mL for 5 μmol peptide) followed by precipitation with cold Et_2_O/pentane (3/1 v/v). Purification by HPLC gives the peptide as a white powder.

^*^Note: We encountered difficulties in the synthesis of PCNA-K164 peptide (UniProtKB - P12004, AA 156-172: ac-DAVVISCAazOrnDGVKFSAS-CONH_2_) following our regular procedure (double couplings 2 × 25 min) on the Syro II. The desired peptide could not be obtained and truncated versions of the peptide were found. To solve this, we synthesized this peptide on an Intavis MutiPep CF automated peptide synthesizer with a real-time UV monitoring enabling the optimization of reaction parameters like deprotection and coupling times during the synthesis. From cycle 13 onwards the coupling times were extended to 2 × 70 min (corresponding to peptide sequence DAVV).

### Synthesis of Ub-Peptide Conjugate

#### Synthesis of Non-hydrolyzable End-Modified Ub-Peptide Conjugates

UbPA and the azidoornithine peptide are each dissolved in warm DMSO at a concentration of 50 mg/mL. 200 μL of the UbPA DMSO stock (10 mg) is added to 15 mL 8M urea, 100 mM phosphate buffer pH 7, followed by addition of 1.2 equiv of the azidoornithine peptide. To the resulting solution is added 600 μL of a freshly prepared CuAAC catalyst solution. This is made by first mixing 200 μL of a 25 mg/mL CuSO_4_·5H_2_O solution in MQ with 200 μL of a 120 mg/mL sodium ascorbate solution in MQ, affording a dark brown solution. Upon addition of 200 μL of a 50 mg/mL Tris((1-benzyl-4-triazolyl)methyl)amine (TBTA) solution in CH_3_CN (Zhou and Fahrni, 2004), a colorless CuAAC catalyst solution is obtained. After the reactions are finished, as judged by LC-MS (~1 h), the reaction is quenched by the addition of 1.5 mL of 0.5 M EDTA, pH 7.0 and the crude product purified by HPLC.

#### Synthesis of Native Iso-Peptide Linked Ub-Peptide Conjugates

A solution of UbSEt (50 mg/mL) in 6M guanidine hydrochloride (GdnHCl), 0.15 M sodium phosphate buffer, pH 7 and 250 mM MPAA is mixed with a solution of the γ-thiolysine peptide mutant (1.5 equiv, 50 mg/mL) in 6M GdnHCl, 0.2 M sodium phosphate buffer, pH 7 and 250 mM 4-mercaptophenylacetic acid (MPAA). After incubating the mixture overnight at 37°C, tris(2-carboxyethyl)phosphine (TCEP) is added to reduce the MPAA disulfide and the crude purified by HPLC.

### Antigen Crosslinking

For coupling the synthesized antigen to carrier protein, several methods can be considered (Yokoyama et al., 2013; Greenfield, 2014; Ossipow and Fischer, 2014; Holzlöhner and Hanack, 2017), depending on the sequence of the peptide and the compatibility with the chemical synthesis of the Ub-peptide conjugates. Here we describe a general crosslinking protocol that uses glutaraldehyde for amine-to-amine coupling; the internal lysines on Ub provide several amines for efficient coupling to the carrier protein. To avoid selecting clones specific for the crosslinked conjugate, it is important to perform the screening of hybridomas with non-crosslinked peptide-Ub conjugates, as explained in more detail below in section 4. To use the synthesized Ub-peptide conjugate for immunization, prior to injection they are crosslinked to Keyhole limpet hemocyanin (KLH) or Blue Carrier Protein (BCP), or a mix of both carrier proteins. Generally, reacting equal mass amounts of Ub-peptide conjugate and carrier protein will achieve sufficient molar excess. Dissolving the Ub-peptide conjugate must be performed with great care to avoid precipitation of the conjugate. First, dissolve the peptide in warm DMSO at a concentration of 40 mg/mL. Then add the DMSO solution dropwise to PBS buffer (phosphate-buffered saline containing 100 mM phosphate buffer), while gently shaking the mixture. Next buffer exchange and concentrate the solution to a final concentration of 10 mg/mL Ub-peptide in PBS. Combine one volume carrier protein (10 mg/mL in PBS) with one volume Ub-peptide conjugate (10 mg/mL in PBS) and mix. Add 1/200 volume of glutaraldehyde (5% in H_2_O) and mix briefly on a vortex. Incubate for 5 min at room temperature and repeat the addition of glutaraldehyde three times. Incubate 30 min on ice. Add 1/10 volume 1M glycine pH 8.5 and incubate for 5 min at room temperature. Dialyze overnight against PBS. Adjust the concentration to the equivalent of 2 mg/mL Ub-peptide conjugate. Store at −20°C. For primary and secondary immunizations of 10 mice, 4 mg Ub-peptide conjugate (400 μl of a 10 mg/mL solution) was combined with 4 mg carrier protein (400 μl of a 10 mg/mL solution). After addition of glutaraldehyde and during dialysis, some aggregation may occur. The immunizations are performed with the mix of soluble and insoluble conjugates since both have been reported to be immunogenic.

### Immunization

Immunization of animals, monitoring the immune response and antibody production of selected clones by ELISA, production of hybridomas, and subcloning and isolation of specific hybridomas is frequently outsourced to a third party specialized in antibody development using proprietary protocols. All hybridomas described in this study were delivered by ThermoFisher Scientific (Life Technologies). All animals used by Thermo Scientific are assured by the Office of Laboratory Animal Welfare. Upon request additional accreditations can be provided. Here we focus on Ub-peptide conjugate synthesis and considerations for validation of site-specific Ub antibodies. Preferably, multiple mice (10 or more) and different genetic mouse backgrounds are immunized to maximize the diversity of the antibody repertoire and immune response. Following primary immunization, the animals are boosted several times until the response stabilizes.

### Antibody Production and Purification

Following hybridoma clone selection and expansion, the yH2B-K123ub1 and candidate PCNA-K164ub1 antibodies were produced and purified in house using the following protocol. Hybridoma clones are initially expanded in flasks in Dulbecco's Modified Eagle Medium (DMEM) or Iscove's Modified Dulbecco's Medium (IMDM) with L-glutamine containing Penicillin and 15-20% fetal calf serum (FCS). The percentage of serum is dropped by 1–2% on every passage until a concentration of 3–6% FCS is reached to minimize the amount of bovine IgG in the media. The final expansion is performed in roller bottles in which the cells are grown for 10–12 days until ~50% of the cells die. The supernatant is collected and debris and cells are removed by centrifugation. The cleared supernatants are stored at 4°C in the presence of 0.02% sodium azide. The pooled supernatants are concentrated and dialyzed using an artificial kidney (Fresenius Medical Care Hemoflow F40S, Polysulfone Capillary Dialyzers; following the manufacturer's instructions) in MES buffer (25 mM 2N-morpholino ethanesulfonic acid, pH 5,5, set with 5 M NaOH), reaching a 20-fold concentration of proteins. The IgGs are purified over ABx resin (Bakerbond ABx prep scale 40 μm JT Baker 7269-00) on a fast protein liquid chromatography (FPLC) system (NGC Bio-Rad) (Ross et al., 1987; Chen et al., 1988) and eluted with an ammonium sulfate gradient (0–100% 500 mM (NH_4_)_2_SO_4_ + 20 mM KH_2_PO_4_ pH6.7) to avoid pH shock (compared with classical ProtA and ProtG methods). Since every antibody has a different elution pattern, the peak fractions are identified by SDS-PAGE analysis for the presence of the IgG light and heavy chains, and subsequently pooled and dialyzed against PBS to remove the ammonium sulfate and azide. Antibodies are stored at 4 degrees for short term use, or frozen in aliquots.

## Anticipated Results

### Ub-Peptide Conjugates and Antigen Crosslinking

**Figure 3:**
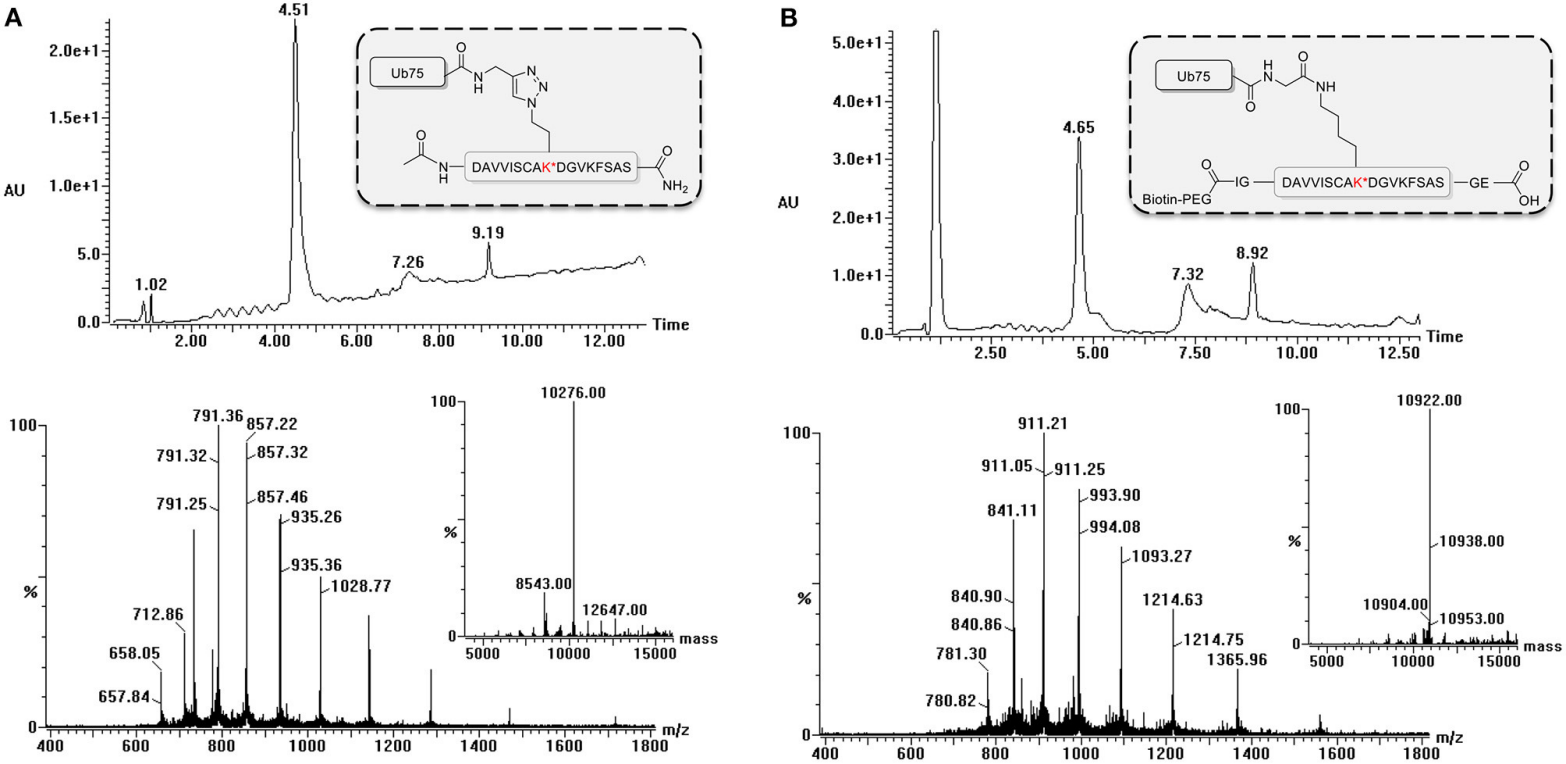
LC-MS analysis of ubiquitinated polypeptides. Example of LC-MS analysis based on PCNA-K164 (UniProtKB P12004). Diode Array chromatogram (top), MS spectrum of peak (bottom) and Deconvoluted mass of product peak (insert) of **(A)** the non-hydrolyzable Ub-conjugate and **(B)** the isopeptide linked Ub-conjugate.

During the synthesis of the Ub-peptide conjugates, traces of monoUb precursors can remain present. Therefore, the purification of the proteolytically-stable Ub-polypeptide conjugates should be performed with great care, in particular as these are used for the immunization. Typically, purification by HPLC is sufficient to remove these traces as can be judged via LC-MS analysis (Figure 3). If difficulties are encountered, an additional size exclusion, or cation purification can be performed (El Oualid et al., 2010). To use the synthesized Ub-peptide conjugates for immunization, prior to injection they are crosslinked to carrier proteins. A crucial step here involves dissolving the Ub-conjugates in warm DMSO. When this step is overlooked and buffer added immediately to Ub, the protein will crash out of solution.

### Immunization of Mice, Generation of Hybridomas, and Isolation of Stable Clones

**Figure 4:**
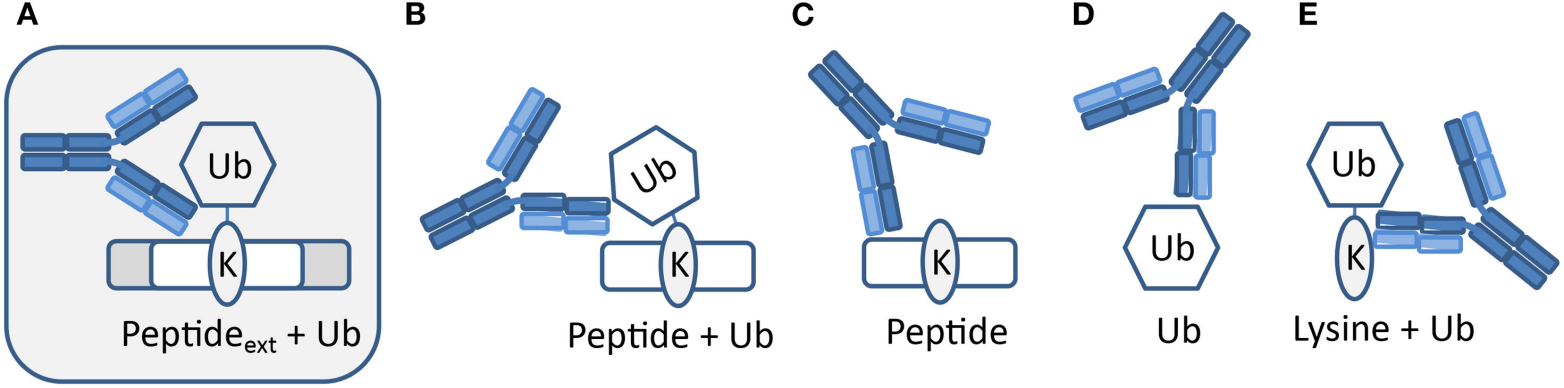
Expected types of antibodies upon immunization with peptide-Ub conjugates. Polyclonal serum after immunization with a non-hydrolyzable end-modified Ub-peptide conjugate will typically contain a mix of antibodies. **(A)** Antibodies recognizing the site-specific ubiquitin on the native protein can be identified using an extended version of the antigen used for immunization. Other antibodies might recognize **(B)** an epitope that includes the terminus of the immunization antigen and hence these antibodies will not recognize ubiquitin in the native extended protein, **(C)** the peptide independent of Ub, **(D)** Ub independent of the peptide, and **(E)** a K-Ub conjugate independent of the peptide context.

**Figure 5:**
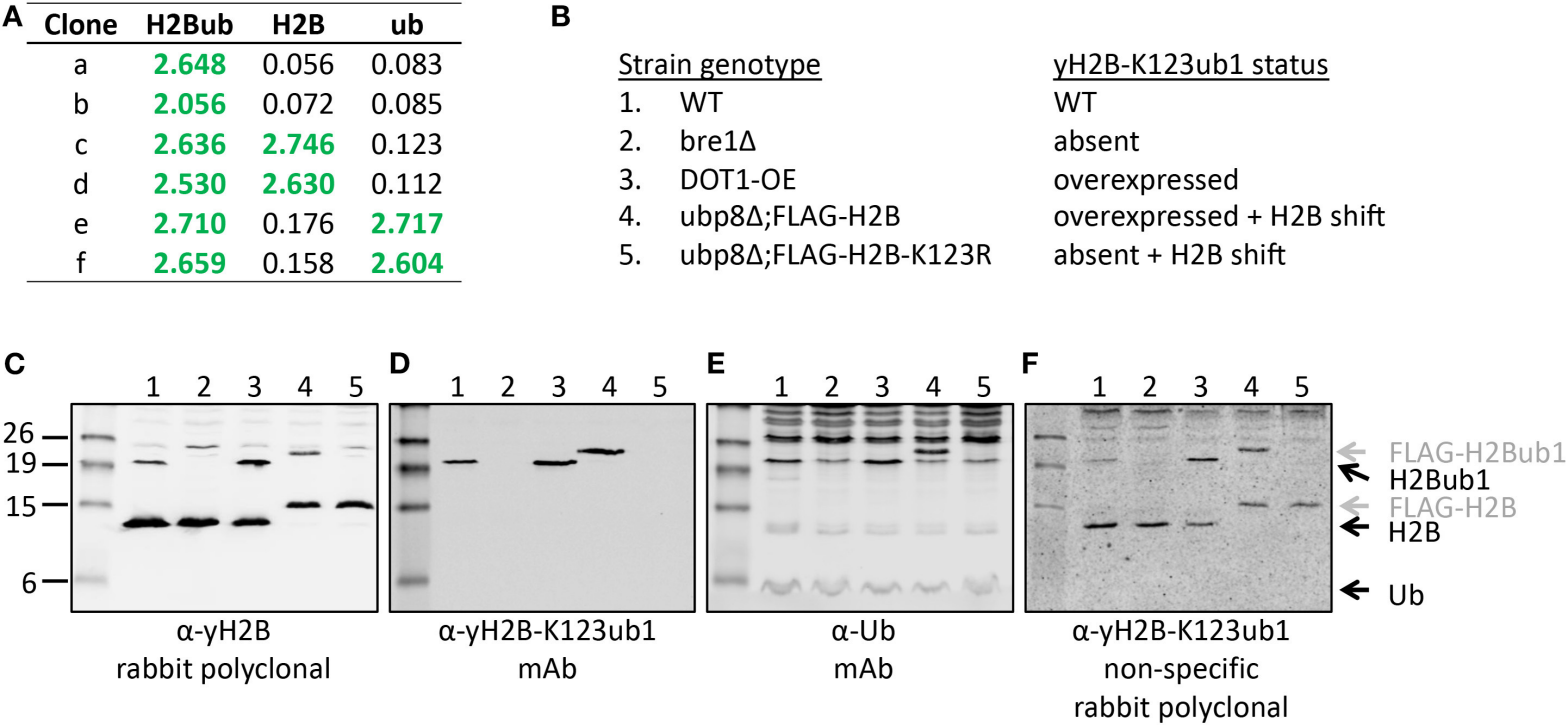
Validation of the site-specific antibody against yeast histone H2B-K123ub1 using engineered cell lines. **(A)** Example of ELISA screening of supernatants of hybridoma cultures that identified clones specific for the H2B-K123ub1 conjugate (#a,b) and clones non-specifically recognizing the H2B peptide (#c,d) or ubiquitin (#e,f). **(B–F)** Following ELISA screens, antibodies produced from selected hybridomas against yH2B-K123ub were validated by immunoblot analysis using whole-cell extracts of a panel of engineered yeast strains. The panel includes (1) WT (normal H2B-K123ub1), (2) bre1Δ (no H2B-K123ub1), (3) DOT1-OE (more H2B-K123ub1), (4) ubp8Δ; FLAG-H2B (more H2B-K123ub1 and size shift due to FLAG-tag), (5) ubp8Δ; FLAG-H2B-K123R (no H2B-K123ub1 and H2B size shift due to FLAG-tag). The strains (BY4741, NKI4558, NKI4553, NKI2563, NKI2564) and protocols have been described previously (Vlaming et al., 2014; van Welsem et al., 2018). The antibodies and polyclonal serum indicated are described in the main text and section 2.2.2.

**Figure 6:**
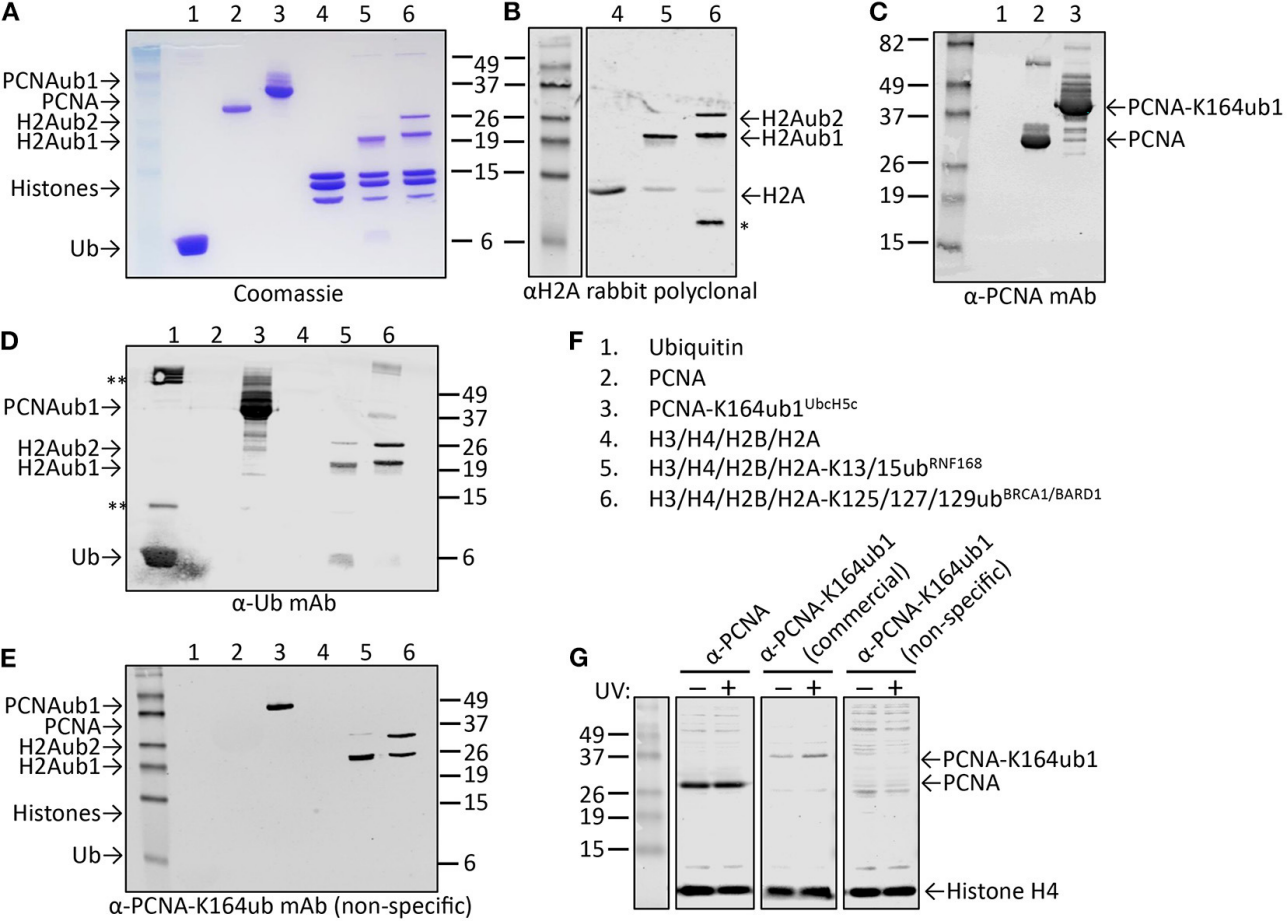
Characterization of candidate site-specific antibody against human PCNA-K164ub1 using recombinant proteins and cell lysates. **(A)** Following ELISA screens, antibodies produced from selected hybridomas against PCNA-K164ub were examined by immunoblot analysis using recombinant proteins that were enzymatically ubiquitinated. The recombinant proteins used are shown on a Coomassie-stained gel. **(B–F)** Immunoblots of recombinant proteins with the indicated antibodies and recombinant proteins. Asterisk indicates degradation product; double asterisk indicates aggregation product. The selected PCNA-K164ub clone produced non-specific antibodies since ubiquitinated H2A was also detected. **(G)** Immunoblot analysis of whole-cell lysates of HEK-293T cells treated or not with 20 J/m^2^ UV for 5 h, confirmed the lack of specificity of the selected clone (non-specific), while a UV-induced PCNA-K164ub1 increase was detected with a commercially available site-specific antibody (CST). Histone H4 was used as a loading control.

The polyclonal serum after immunization with a non-hydrolyzable end-modified Ub-peptide conjugate will typically contain a mix of antibodies, e.g., anti-peptide, anti-ubiquitin, and anti-peptide-Ub (Figure 4). Given the relatively large size of the Ub moiety, a substantial anti-Ub response is expected, which we indeed observed in immunized mice (see below) and rabbits (Figures 5A,E). Nevertheless, it is useful to monitor the general immune response by ELISA to decide which of the mice will be used for hybridoma generation. After a final boost with antigen, spleens of three positive mice are harvested to isolate the antibody-producing cells for cell fusion to generate hybridomas. Hybridoma clones of ~30 96-well plates are screened by ELISA using the native extended Ub-peptide conjugate and peptide and ubiquitin alone to identify clones that specifically recognize the site-specific ubiquitin attachment. Positive and specific clones identified by ELISA that show increased or stable production of specific antibodies (Figure 5A) are also screened by immunoblot analysis with recombinant proteins to confirm that antibodies in these clones recognize the native ubiquitin linkage on the complete protein (Figures 5, 6).

### Design Principles for Immunization- and Screening-Antigens

Since the validation assays are labor intensive, several design principles are recommended in the ELISA screening protocol to eliminate as many false positive clones as possible.

To identify clones that produce Ub site-specific antibodies, hybridoma culture supernatants are screened by ELISA. ELISA screens are performed with polypeptides that are two amino acids longer at the N-terminus and/or C-terminus (depending on the position of the ubiquitination site in the protein) than the antigen to more closely mimic the native protein and to eliminate clones that recognize the N- and/or C terminal ends of the peptide normally not present in the full-length protein. The yH2B peptide was not extended at the C-terminus because it represents the C-terminal end of the native H2B protein.

While immunizations are performed with non-hydrolyzable Ub conjugates, ELISA tests are performed with a native peptide-ubiquitin linkage to avoid the selection of clones that recognize the non-native bond (Figures 2, 4). A specific signal in the ELISA test is a requirement for a specific antibody but it does not guarantee that an antibody will be effective. An example of different ELISA results is shown in Figure 5. In our experience, not all ELISA-positive clones recognized the target in subsequent validation assays. This highlights the importance of validation assays using native substrates.

### Validation of the Antibody

Validation of antibodies is critical but it has often been overlooked. Fortunately, there are many recent initiatives to improve the guidelines for quality control and thereby the value of the antibodies and the results that are generated with them (Egelhofer et al., 2010; Älgenäs et al., 2014; Bradbury and Plückthun, 2015; Kungulovski et al., 2015; Marcon et al., 2015; Rothbart et al., 2015; Uhlen et al., 2016; Guillemette et al., 2017; Edfors et al., 2018; Venkataraman et al., 2018; Weller, 2018; Marx, 2019). Validation of site-specific Ub-antibodies is very important given the abundance of ubiquitin modifications in the cell and the complexity of the epitope. Here we describe several examples of quality control experiments for site-specific Ub-antibodies. During the initial screening and validation tests, only a small amount of antibody is typically available, limiting the number of experiments that can be performed. On selected clones that give a positive and specific response by ELISA, immunoblots can be performed as a secondary screening to identify those antibodies that recognize the full-length ubiquitin conjugate but in a denatured form. Based on the combined screening results, clones are selected for expansion and purification of secreted immunoglobulins, as described above. It is also useful to consider the immunoglobulin class and subclass in the context of the downstream applications of the antibodies. If the peptides are synthesized with a biotin-moiety, this allows for using the conjugates in assays such as Surface Plasmon Resonance (SPR) to determine the binding properties of the antibodies in more detail.

A convenient method for validation of purified antibodies is the analysis of the full-length modified proteins by immunoblot. Here we show two examples, taking advantage of different sets of reagents. Figure 5 summarizes the screening and validation results of the yH2B-K123ub antibody. Yeast strains, antibodies and protocols to detect H2BK123ub1 by immunoblot assays have been described previously (Vlaming et al., 2016, 2019; van Welsem et al., 2018). In the ELISA screens, clones were identified that produced antibodies recognizing H2B-K123ub1 and not recognizing unmodified H2B or free Ub. We also observed clones that recognized Ub non-specifically (Figures 5A,E and see below), one of which we selected for expansion and antibody production. The clones with site-specific signals in ELISA (e.g., see Figure 5A) were examined on immunoblots with yeast strains in which yH2B-K123ub was wild-type, absent, increased, and shifted by the addition of a short epitope tag on H2B (Figures 5B–F). When using cell extracts, this has the added benefit that non-specific reactions to other cellular—ubiquitinated–proteins can be identified. Together, the signals observed in this panel of strains demonstrated the specificity of the antibody for yH2B-K123ub in yeast. Importantly, the specificity was subsequently confirmed by chromatin-immunoprecipitation assays, which demonstrated loss of signal in strains lacking yH2B-K123ub and gain of signal in strains with excess of it (van Welsem et al., 2018; Vlaming et al., 2019). Of note, injection of the yH2B-K123ub conjugate in rabbits resulted in a strong polyclonal response including non-specific antibodies against H2B and Ub (e.g., see Figure 5F), highlighting the need for selecting monoclonal antibodies. This is in contrast with antibodies against smaller modifications such as methylation, phosphorylation, or acetylation, for which specific polyclonal antisera have been successfully developed.

While cell extracts provide a powerful system to determine antibody specificity, it is not always straightforward or even possible to modulate the levels of the site-specific ubiquitination in cells, for example due to redundant enzyme activities, compensating ubiquitination and deubiquitination activities, fitness defects due to ubiquitination changes, or insufficient knowledge of the enzymes involved. However, in some cases the epitope in cells can be abolished by mutation of the target lysine to arginine, as we previously demonstrated for PCNA-K164 (Krijger et al., 2011). Another limitation is that for some sites, the endogenous, cellular levels may be too low to allow for detection during the screening and validation without an enrichment procedure. A powerful alternative to using cell extracts is the use of recombinant proteins and installing the ubiquitination with chemical and/or enzymatic methods. Figures 6A–F summarizes the results of the screening and validation phase of an antibody against ubiquitinated lysine 164 on human PCNA (PCNA-K164ub). Clones recognizing the PCNA-K164ub1 and not recognizing unmodified PCNA peptide or free Ub in ELISA were examined on immunoblots using recombinant proteins (Figures 6A–F). Recombinant human PCNA was purified and ubiquitinated enzymatically as described previously (Hibbert and Sixma, 2012). Recombinant nucleosomes enzymatically ubiquitinated at histone H2A-K13/15 by RNF168 and H2A-K125/127129 by BRCA1-BARD1 (Zhu et al., 2011; Mattiroli et al., 2012, 2014; Kalb et al., 2014; Uckelmann et al., 2018; Dharadhar et al., 2019; Horn et al., 2019) were included as controls (Figures 6A–C). Among the analyzed clones we observed antibodies recognizing PCNA and Ub alone. We also identified antibodies recognizing PCNA-K164ub1 but not unmodified full-length PCNA or Ub (Figure 6D). However, the selected clones appeared not to be site-specific because enzymatically ubiquitinated histone H2A on lysine 13/15 or 125/127/129 was also detected (Figure 6D). These results suggest that the selected clone produces antibodies recognizing the ubiquitinated lysine in PCNA but not specifically in the context of PCNA. Indeed, in cellular extracts, these antibodies detected a range of proteins, further indicating the lack of specificity for PCNA (Figure 6G). Therefore, when using recombinant proteins for validation, it is important to include independent ubiquitinated proteins in the analysis. It may also be beneficial to include independent peptide-Ub conjugates in the ELISA screens for selection and monitoring of hybridoma clones. Antibodies recognizing lysine-Ub conjugates provide powerful reagents for detecting poly- and mono-ubiquitinated proteins in cells and for affinity purification (Fujimuro and Yokosawa, 2005; Fulzele and Bennett, 2018; van Wijk et al., 2019). The clones we describe here potentially expand the toolbox for detecting protein ubiquitination but it will be important to determine their binding properties to the different types of linkages in more detail (Fujimuro et al., 1994; Newton et al., 2008, 2012; Matsumoto et al., 2010, 2012), and to do so in the assays of interest and not just on immunoblots.

## Discussion

Whereas polyclonal antibodies can specifically recognize sites carrying small post-translational modifications, a polyclonal response against ubiquitinated targets will lead to a mix of antibodies recognizing the ubiquitinated site, the unmodified peptide, any ubiquitinated lysine, and ubiquitin alone, as we experienced in yH2B-K123ub1 and PCNA-K164ub1 antibody screens. Therefore, the generation of site-specific ubiquitin antibodies will typically require the development of monoclonal antibodies. The antibodies we describe here were generated in mice. It has recently become possible and more popular to develop monoclonal antibodies in rabbits using different technologies (Weber et al., 2017). Since rabbits have highly distinctive antibody repertoires, this may offer powerful additional opportunities. Another powerful alternative is the generation of single-chain antibodies or nanobodies in llamas. Nanobodies recognizing linear and conformational epitopes can be obtained, which may be of special relevance for ubiquitinated conjugates (https://instruct-eric.eu/platform/nanobody-discovery). The synthesis of Ub-peptide conjugates and the suggestions for design and validation experiments are also applicable to rabbit monoclonals and nanobodies. However, with larger animals, more antigen is typically needed for immunization. Using automated linear solid phase peptide synthesis and the chemical synthesis ligation technologies described, we are able to fully synthesize site-selective ubiquitinated peptides in a native conformation as well as proteolytically stable non-hydrolyzable derivates. This synthetic approach enables the specific incorporation of desired tags, labels, and mutations in high yields with high purities. Access to these well-defined ubiquitinated peptides, is of utmost importance in these types of studies.

The complexity and size of the site-specific ubiquitination epitope also means that stringent screening and validation strategies are required for the selection of appropriate antibodies. Initial screening of hybridoma clones is typically performed by ELISA. Recent advances in next generation sequencing offer powerful alternative strategies for antibody selection by identifying the genes encoding the antibody of interest. These methods are based on identifying clones dominating the total plasma cell repertoire following secondary immunization (Haessler and Reddy, 2014; Parola et al., 2018). However, when immunizations are performed with ubiquitinated peptides, the expanding clones will represent antibodies against peptide, ubiquitin, and non-specific lysine-ubiquitin conjugates and only a minority of the clones will produce site-specific antibodies (e.g., see Figures 5, 6). Therefore, more targeted screening methods are still required when complex antigens are used and when site-specific ubiquitin antibodies are wanted. Validation of the antibodies by immunoblot analysis, as we describe in Figures 5, 6, provides a convenient way of validating the specificity and sensitivity of the antibody. This is especially useful during the early stages of validation, when limiting amounts of antibody are available from the culture supernatants of the clones. However, this assay does not predict how well the antibody will work for other applications. Similarly, a specific signal on immunoblots does not guarantee that the antibody is specific in other assays. Therefore, once the antibody has been produced at larger scales, it should always be carefully tested in the application of interest. Validation assays, whether using cells or recombinant proteins, should include several critical negative controls, including the non-modified protein, free ubiquitin, and independent ubiquitinated proteins. Fortunately, the expanding tool sets for genome editing and *in vitro* ubiquitination allow for the generation of many powerful control settings for future antibody development. With regard to ubiquitination sites on chromatin, one protein of special interest is histone H2A or the histone variant H2AX (Mattiroli and Sixma, 2014; Du et al., 2019; Marsh and Dickson, 2019). H2A is ubiquitinated at several sites by specific enzyme complexes and each with a specific role in DNA damage response, DNA repair, and gene silencing (Mattiroli and Sixma, 2014; Marsh and Dickson, 2019). We hope that our protocols will be of use for others in the community to develop novel site-specific antibodies against these and other important epitopes.

## Data Availability Statement

All datasets generated for this study are included in the article/supplementary material.

## Ethics Statement

Immunization of animals, monitoring the immune response and antibody production of selected clones by ELISA, production of hybridomas, and subcloning and isolation of specific hybridomas is frequently outsourced to a third party specialized in antibody development using proprietary protocols. All hybridomas described in this study were delivered by ThermoFisher Scientific (Life Technologies). All animals used by Thermo Scientific are assured by the Office of Laboratory Animal Welfare. Upon request additional accreditations can be provided.

## Author Contributions

IK, MM, FE, HO, and FL contributed conception and design of the study. IK and TW performed quality control experiments and coordinated the studies. MM was responsible for chemical synthesis. MU generated recombinant proteins. JW purified monoclonal antibodies. AS performed quality control experiments under supervision of HJ. IK, MM, FE, HO, and FL wrote sections of the manuscript. All authors contributed to manuscript revision, read and approved the submitted version.

### Conflict of Interest

FL, HO, and FE are entitled to royalties resulting from antibody licensing. FE and HO declare competing financial interests as co-founders and shareholders of UbiQ Bio BV. The remaining authors declare that the research was conducted in the absence of any commercial or financial relationships that could be construed as a potential conflict of interest.
